# Supplementary material for: Functional groups of Afrotropical EPT (Ephemeroptera, Plecoptera and Trichoptera) as bioindicators of semi-urban pollution in the Tsitsa River Catchment, Eastern Cape, South Africa
Source: PeerJ. 2022 Dec 15;10:e13970. doi: 10.7717/peerj.13970 (PMC9760020; doi:10.7717/peerj.13970)
Supplement: Supplemental Information 1 [file peerj-10-13970-s001.docx]

Supplementary data.

Table S1: Mean abundance of EPT (Ephemeroptera Plecoptera and Trichoptera) collected during the study period

| **Taxa** | **SG 1** | **SG2** | **SG3** |
| --- | --- | --- | --- |
| *Acanthiops* sp. | 11.42 | 0.00 | 1.58 |
| *Acanthiops tsitsa* | 23.00 | 9.75 | 5.75 |
| *Adenophlebia auriculata* | 8.08 | 0.00 | 9.42 |
| *Adenophlebia* sp. | 6.75 | 0.00 | 4.58 |
| *Afronurus bernardi* | 5.08 | 0.00 | 0.00 |
| *Afronurus* sp. | 14.92 | 0.00 | 2.08 |
| *Aphenicarca* sp. | 1.17 | 0.00 | 2.42 |
| *Baetis* sp. | 34.25 | 32.88 | 32.25 |
| *Caenis* sp. | 47.58 | 66.88 | 3.00 |
| *Elassoneuria* sp.. | 73.33 | 2.25 | 41.50 |
| *Euthraulus* sp. | 22.33 | 53.63 | 7.50 |
| *Hydropsyche* sp. | 1.33 | 0.00 | 0.33 |
| *Oligoneuriopsis lawrencei* | 0.00 | 0.00 | 0.75 |
| *Oligoneuriopsis* sp. | 0.08 | 0.00 | 0.00 |
| *Prosopistoma amanzamanya* | 8.42 | 10.38 | 6.17 |
| *Pseudocloeon glaucum* | 19.83 | 6.13 | 12.00 |
| *Pseudocloeon piscis*. | 29.25 | 29.00 | 42.25 |
| *Pseudocloeon* sp. | 11.83 | 7.63 | 5.08 |
| *Pseudocloeon vinosum*. | 43.58 | 0.13 | 1.42 |
| *Tricoryhus* sp. | 43.58 | 0.13 | 1.42 |
| *Cheumatopsyche* sp. | 15.92 | 17.25 | 6.67 |
| *Macrostenum* *capense* | 20.50 | 11.88 | 12.08 |
| *Cheumatopsyche* *thomasseti* | 13.33 | 19.88 | 19.08 |
| *Chematopsyche* *afra* | 8.00 | 12.50 | 1.67 |

Table S2: Raw data of Ephemeroptera, Plecoptera and Trichoptera taxa collected during the study in the Tistisa River between 2016 – 2017.

| SG | Acanthiops sp. | Acanthiops tsitsa | Adenophlebia auriculata | Adenophlebia sp. | Afronurus bernadi | Afronurus sp. | Apheni sp. | Baetis sp | Caenis sp. | Chematopsyche afra | Cheumatopsyhe sp. | Cheumatopsyche thomasseti | Elassoneuria sp. |
| --- | --- | --- | --- | --- | --- | --- | --- | --- | --- | --- | --- | --- | --- |
| SG1_W | 20 | 0 | 0 | 0 | 0 | 35 | 0 | 0 | 34 | 0 | 0 | 2 | 50 |
| SG1_SP | 0 | 6 | 0 | 0 | 0 | 0 | 1 | 3 | 43 | 0 | 0 | 2 | 188 |
| SG1_SU | 0 | 22 | 0 | 0 | 0 | 2 | 8 | 37 | 18 | 5 | 4 | 8 | 30 |
| SG1_A | 11 | 0 | 0 | 0 | 0 | 1 | 5 | 18 | 0 | 0 | 37 | 23 | 21 |
| SG1_W1 | 0 | 139 | 0 | 0 | 0 | 42 | 0 | 0 | 155 | 0 | 70 | 0 | 0 |
| SG1_SP1 | 0 | 109 | 0 | 0 | 0 | 25 | 0 | 0 | 69 | 0 | 0 | 0 | 259 |
| SG1_SU1 | 20 | 0 | 0 | 0 | 7 | 0 | 0 | 39 | 15 | 0 | 0 | 0 | 16 |
| SG1_A1 | 26 | 0 | 40 | 0 | 0 | 22 | 0 | 134 | 5 | 87 | 40 | 56 | 25 |
| SG1_W2 | 1 | 0 | 17 | 81 | 9 | 30 | 0 | 0 | 139 | 0 | 0 | 1 | 75 |
| SG1_SP2 | 13 | 0 | 0 | 0 | 38 | 0 | 0 | 7 | 73 | 0 | 0 | 1 | 175 |
| SG1_SU2 | 20 | 0 | 0 | 0 | 7 | 0 | 0 | 39 | 15 | 4 | 0 | 0 | 16 |
| SG1_A2 | 26 | 0 | 40 | 0 | 0 | 22 | 0 | 134 | 5 | 0 | 40 | 67 | 25 |
| SG2_W | 0 | 0 | 0 | 0 | 0 | 0 | 0 | 0 | 134 | 0 | 0 | 1 | 0 |
| SG2_SP | 0 | 0 | 0 | 0 | 0 | 0 | 0 | 0 | 88 | 0 | 0 | 0 | 1 |
| SG2_SU | 0 | 3 | 0 | 0 | 0 | 0 | 0 | 99 | 25 | 0 | 0 | 1 | 0 |
| SG2_A | 0 | 0 | 0 | 0 | 0 | 0 | 0 | 73 | 0 | 0 | 23 | 0 | 0 |
| SG2_W1 | 0 | 0 | 0 | 0 | 0 | 0 | 0 | 0 | 154 | 0 | 0 | 2 | 17 |
| SG2_SP1 | 0 | 0 | 0 | 0 | 0 | 0 | 0 | 7 | 75 | 98 | 10 | 23 | 0 |
| SG2_SU1 | 0 | 73 | 0 | 0 | 0 | 0 | 0 | 74 | 59 | 1 | 105 | 54 | 0 |
| SG2_A1 | 0 | 2 | 0 | 0 | 0 | 0 | 0 | 10 | 0 | 1 | 0 | 78 | 0 |
| SG3_W | 19 | 0 | 0 | 15 | 0 | 0 | 0 | 44 | 0 | 4 | 3 | 12 | 119 |
| SG3_SP | 0 | 3 | 0 | 0 | 0 | 5 | 0 | 53 | 0 | 0 | 0 | 1 | 72 |
| SG3_SU | 0 | 0 | 0 | 2 | 0 | 11 | 0 | 23 | 0 | 0 | 22 | 80 | 122 |
| SG3_A | 0 | 0 | 13 | 0 | 0 | 1 | 0 | 0 | 0 | 0 | 0 | 56 | 2 |
| SG3_W1 | 0 | 0 | 0 | 0 | 0 | 3 | 0 | 0 | 7 | 12 | 0 | 1 | 8 |
| SG3_SP1 | 0 | 0 | 0 | 0 | 0 | 0 | 0 | 0 | 15 | 0 | 0 | 1 | 0 |
| SG3_SU1 | 0 | 0 | 0 | 0 | 0 | 3 | 9 | 3 | 8 | 0 | 0 | 1 | 39 |
| SG3_A1 | 0 | 1 | 0 | 0 | 0 | 1 | 18 | 17 | 5 | 0 | 0 | 1 | 0 |
| SG3_W2 | 0 | 0 | 26 | 0 | 0 | 1 | 0 | 74 | 0 | 0 | 0 | 0 | 53 |
| SG3_SP2 | 0 | 0 | 66 | 9 | 0 | 0 | 0 | 0 | 0 | 4 | 19 | 67 | 15 |
| SG3_SU2 | 0 | 11 | 0 | 29 | 0 | 0 | 1 | 97 | 0 | 0 | 19 | 1 | 39 |
| SG3_A2 | 0 | 54 | 8 | 0 | 0 | 0 | 1 | 76 | 1 | 0 | 17 | 8 | 29 |

Table S2 continuation…

| SG | Euthraulus sp. | Hydropsyche sp. | Macrostenum capense | Oligonueriopsis lawrenceis | Oligochaeta sp. | Prosopistoma amanzamanya | Pseudocloeon glaucum | Pseudocloeon piscis. | Pseudocloeon sp. | Pseudocloeon vinosum | Tricorythus sp. |
| --- | --- | --- | --- | --- | --- | --- | --- | --- | --- | --- | --- |
| SG1_W | 0 | 0 | 1 | 0 | 0 | 0 | 68 | 23 | 0 | 0 | 0 |
| SG1_SP | 47 | 0 | 1 | 0 | 0 | 0 | 0 | 53 | 0 | 90 | 90 |
| SG1_SU | 3 | 16 | 23 | 0 | 1 | 56 | 0 | 0 | 0 | 32 | 32 |
| SG1_A | 1 | 0 | 34 | 0 | 0 | 30 | 0 | 0 | 4 | 104 | 104 |
| SG1_W1 | 0 | 0 | 90 | 0 | 0 | 0 | 55 | 79 | 0 | 8 | 8 |
| SG1_SP1 | 96 | 0 | 1 | 0 | 0 | 0 | 2 | 36 | 0 | 23 | 23 |
| SG1_SU1 | 0 | 0 | 56 | 0 | 0 | 0 | 30 | 0 | 59 | 17 | 17 |
| SG1_A1 | 33 | 0 | 0 | 0 | 0 | 7 | 5 | 39 | 9 | 56 | 56 |
| SG1_W2 | 32 | 0 | 0 | 0 | 0 | 0 | 26 | 54 | 0 | 101 | 101 |
| SG1_SP2 | 19 | 0 | 0 | 0 | 0 | 1 | 17 | 28 | 2 | 19 | 19 |
| SG1_SU2 | 4 | 0 | 0 | 0 | 0 | 0 | 30 | 0 | 59 | 17 | 17 |
| SG1_A2 | 33 | 0 | 40 | 0 | 0 | 7 | 5 | 39 | 9 | 56 | 56 |
| SG2_W | 50 | 0 | 0 | 0 | 0 | 0 | 7 | 9 | 0 | 0 | 0 |
| SG2_SP | 16 | 0 | 0 | 0 | 0 | 0 | 0 | 8 | 10 | 0 | 0 |
| SG2_SU | 36 | 0 | 0 | 0 | 0 | 7 | 0 | 6 | 0 | 1 | 1 |
| SG2_A | 3 | 0 | 0 | 0 | 0 | 12 | 0 | 0 | 25 | 0 | 0 |
| SG2_W1 | 108 | 0 | 0 | 0 | 0 | 0 | 36 | 169 | 0 | 0 | 0 |
| SG2_SP1 | 61 | 0 | 6 | 0 | 0 | 0 | 0 | 5 | 0 | 0 | 0 |
| SG2_SU1 | 21 | 0 | 0 | 0 | 0 | 29 | 4 | 18 | 26 | 0 | 0 |
| SG2_A1 | 134 | 0 | 89 | 0 | 0 | 35 | 2 | 17 | 0 | 0 | 0 |
| SG3_W | 19 | 0 | 1 | 0 | 0 | 0 | 22 | 59 | 0 | 1 | 1 |
| SG3_SP | 4 | 0 | 2 | 0 | 0 | 0 | 18 | 100 | 11 | 0 | 0 |
| SG3_SU | 8 | 0 | 40 | 0 | 0 | 0 | 0 | 48 | 0 | 0 | 0 |
| SG3_A | 0 | 0 | 0 | 0 | 0 | 34 | 0 | 0 | 11 | 0 | 0 |
| SG3_W1 | 1 | 0 | 0 | 0 | 0 | 0 | 65 | 44 | 0 | 0 | 0 |
| SG3_SP1 | 1 | 0 | 0 | 0 | 0 | 0 | 5 | 60 | 0 | 0 | 0 |
| SG3_SU1 | 12 | 4 | 0 | 0 | 0 | 0 | 0 | 3 | 11 | 0 | 0 |
| SG3_A1 | 25 | 0 | 0 | 5 | 0 | 27 | 12 | 11 | 22 | 15 | 15 |
| SG3_W2 | 1 | 0 | 0 | 0 | 0 | 0 | 7 | 21 | 0 | 0 | 0 |
| SG3_SP2 | 0 | 0 | 12 | 0 | 0 | 1 | 14 | 73 | 3 | 0 | 0 |
| SG3_SU2 | 0 | 0 | 56 | 0 | 0 | 12 | 1 | 52 | 0 | 0 | 0 |
| SG3_A2 | 19 | 0 | 34 | 4 | 0 | 0 | 0 | 36 | 3 | 1 | 1 |

Table S3: Fuzzy coded Ephemeroptera, Plecoptera and Trichoptera taxa collected during the study period (2016 – 2017) in the Kat River.

| Taxon | FI_F | GRA | SHR | PRE | CG |
| --- | --- | --- | --- | --- | --- |
| Acanth sp. | 0 | 0 | 0 | 0 | 0 |
| Acanth tsi. | 0 | 0 | 0 | 0 | 0 |
| Adeno auri. | 0 | 0 | 0 | 0 | 3 |
| Adeno sp. | 0 | 0 | 0 | 0 | 4 |
| Afro bern | 0 | 0 | 0 | 0 | 4 |
| Afro sp. | 0 | 0 | 0 | 0 | 5 |
| Apheni sp. | 0 | 0 | 0 | 3 | 0 |
| Baet sp | 0 | 0 | 0 | 2 | 0 |
| Caen sp. | 0 | 0 | 0 | 0 | 2 |
| Chem. Afra | 5 | 0 | 0 | 0 | 0 |
| Chem. Th | 5 | 0 | 0 | 0 | 0 |
| Cheum sp. | 5 | 0 | 0 | 0 | 0 |
| Elass fl. | 0 | 0 | 0 | 0 | 1 |
| Euth sp. | 1 | 0 | 1 | 0 | 2 |
| Hydrop sp. | 5 | 0 | 0 | 0 | 0 |
| Macrostenum capense | 5 | 0 | 0 | 0 | 0 |
| Oligo law. | 0 | 0 | 0 | 0 | 0 |
| Oligo sp | 0 | 0 | 0 | 0 | 5 |
| Proso ama. | 0 | 0 | 0 | 0 | 3 |
| Pseudo glau. | 0 | 0 | 1 | 0 | 3 |
| Pseudo pisc. | 0 | 0 | 1 | 0 | 3 |
| Pseudo sp. | 0 | 0 | 0 | 0 | 3 |
| Pseudo vin. | 0 | 0 | 1 | 0 | 3 |
| Trico sp. | 0 | 0 | 0 | 0 | 4 |

Table S4: Physicochemical variables raw data collected during the study in the Tsitsa River between 2016 – 2017.

| SG | Electric Conductivity | DO | pH | Temperature | NO3 | NO2 | P04 | NH4 | TIN |
| --- | --- | --- | --- | --- | --- | --- | --- | --- | --- |
| SG1_W | 42 | 12.11 | 7.15 | 7.42 | 0.205 | 0.01 | 0.0025 | 0.0025 | 0.05127 |
| SG1_SP | 50 | 10.42 | 8.24 | 23.4 | 0.134 | 3.6 | 0.136 | 0.0025 | 1.12785 |
| SG1_SU | 15 | 7.94 | 7.05 | 23 | 0.43 | 0.13 | 2.73 | 0.59 | 0.59554 |
| SG1_A | 72 | 8.11 | 6.2 | 13 | 0.71 | 0.19 | 0.0025 | 0.15 | 0.334815 |
| SG1_W1 | 49 | 14.21 | 6.93 | 6.27 | 0.17 | 0.0025 | 0.0025 | 0.0025 | 0.041092 |
| SG1_SP1 | 48 | 10.13 | 7.5 | 22.3 | 7.23 | 0.09 | 0.0025 | 0.0025 | 1.65259 |
| SG1_SU1 | 60 | 6.11 | 7.1 | 24.5 | 5.23 | 0.11 | 1.79 | 0.6 | 1.68111 |
| SG1_A1 | 58 | 10.1 | 4.5 | 18 | 1.77 | 0.03 | 0.136 | 0.0025 | 0.4107 |
| SG1_W2 | 38 | 13.43 | 6.28 | 8.31 | 0.238 | 0.032 | 0.0025 | 0.0025 | 0.065425 |
| SG1_SP2 | 44 | 11.9 | 8.19 | 21.2 | 5.11 | 0.067 | 0.0025 | 0.185 | 1.31814 |
| SG1_SU2 | 52 | 5.6 | 8.16 | 23.4 | 0.42 | 0.43 | 2.85 | 0.7 | 0.77015 |
| SG1_A2 | 54 | 8.11 | 5.1 | 18 | 6.13 | 0.05 | 0.109 | 0.0025 | 2.33831 |
| SG2_W | 58 | 11.49 | 7.42 | 5.82 | 0.321 | 0.009 | 0.0025 | 0.0025 | 0.077167 |
| SG2_SP | 77 | 12.15 | 8.6 | 18.6 | 4.9 | 0.152 | 0.109 | 0.0025 | 1.154656 |
| SG2_SU | 50 | 3.88 | 8 | 24 | 3.21 | 0.52 | 3.05 | 0.6 | 1.349766 |
| SG2_A | 61 | 15 | 6.5 | 24 | 7.2 | 0.05 | 0.109 | 0.0025 | 1.642968 |
| SG2_W1 | 79 | 14.31 | 7.66 | 6.73 | 0.321 | 0.001 | 0.0025 | 0.0025 | 0.074507 |
| SG2_SP1 | 74 | 8.39 | 7.92 | 24 | 5.17 | 0.131 | 2.64 | 0.0025 | 1.20748 |
| SG2_SU1 | 50 | 3.96 | 8.15 | 25 | 3.42 | 6.2 | 3.09 | 0.62 | 3.14143 |
| SG2_A1 | 72 | 17.1 | 8.1 | 28 | 10.1 | 0.06 | 2.64 | 0.0025 | 2.30085 |
| SG3_W | 57 | 8.82 | 7.28 | 6.34 | 0.205 | 0 | 0.0025 | 0.0025 | 0.048233 |
| SG3_SP | 74 | 109 | 7.92 | 24 | 0.04 | 0.953 | 0.0025 | 0.0025 | 0.301016 |
| SG3_SU | 88 | 6.8 | 7.02 | 21.79 | 0.25 | 0.41 | 3.63 | 0.73 | 0.749009 |
| SG3_A | 82 | 5.1 | 6.68 | 23.3 | 0.01 | 0.05 | 0.0025 | 0.0025 | 0.02854 |
| SG3_W1 | 46 | 0 | 6.74 | 11.33 | 0.4 | 0 | 0.0025 | 0.0025 | 0.87161 |
| SG3_SP1 | 59 | 21 | 8.12 | 20.1 | 3.86 | 0.16 | 0.0025 | 1.087 | 1.76575 |
| SG3_SU1 | 88 | 6.84 | 7.02 | 21.79 | 2.43 | 0.7 | 3.09 | 0.63 | 1.252144 |
| SG3_A1 | 106 | 8.7 | 8 | 28 | 0.01 | 0.19 | 0.0025 | 0.0025 | 0.55008 |
| SG3_W2 | 106 | 9.05 | 6.75 | 12.33 | 0.404 | 0.0025 | 0.0025 | 0.0025 | 0.09393 |
| SG3_SP2 | 175 | 8.65 | 8.34 | 19.8 | 3.6 | 0.21 | 0.0025 | 0.0025 | 0.87876 |
| SG3_SU2 | 76 | 12.82 | 6.39 | 24 | 2.5 | 0.4 | 3.09 | 0.61 | 1.16069 |
| SG3_A2 | 102 | 0.74 | 6.67 | 23.06 | 0.01 | 0.05 | 0.0025 | 0.0025 | 0.01941 |
